# Debunking the myth of morning skate on game day

**DOI:** 10.3389/fspor.2023.1284613

**Published:** 2023-11-10

**Authors:** Franck Brocherie, Jerome Perez

**Affiliations:** ^1^ Laboratory Sport, Expertise and Performance (EA 7370), French Institute of Sport (INSEP), Paris, France; ^2^ Performance Department, Brûleurs de Loups, Grenoble, France

**Keywords:** ice hockey, priming, superstition, chronobiology, informed decision

## Introduction

In the sport sciences community, some practical questions remain unsounded or unanswered. Among those, morning skate (also called “activation” or “muscular wake-up”; implemented in other sport-specific contexts such as the “morning shoot around” in basketball and baseball) is conducted on game day in most professional ice hockey leagues [*e.g.*, National Hockey League (NHL) in North America, Kontinental Hockey League in Eurasia or National League in Switzerland] without having a clear understanding of its impact on evening game performance.

Such pre-game sessions, consisting in practicing technical and tactical drills for ∼30–45 min in the game-day morning, should be considered as a separate concept from warm-ups (1). The potential effects of morning skate are believed to be related to chronobiology (2), neuromuscular delayed potentiation (3, 4) or simply psychological behavior (5). However, it remains unknown whether the addition and the repetition of such sessions in a dense competitive calendar (*e.g.*, 35 morning skate within the 98 on-ice training sessions and 76 matches performed over 28 weeks in the NHL regular season) is valuable. The aim of this opinion piece is to summarize the empirical point of view emanating from the ice hockey community (*i.e.*, statements from coaches, players and journalists) and to compare it with other sports (in which such morning activity is conducted) or exercise-based scientific evidence in order to help practitioners involved in ice hockey in their decisional process to conduct or not such morning session on game day.

## Empirical background of the morning skate

The reasons why the morning skate was introduced (primarily in North America) are multiple. Historically, it seems that the game-day morning skate originated from the 1940s when Toronto Maple Leafs’ players felt the need to test the sharpening of their skate blades following troubles observed during a previous game (6). While others reported less technical reasons (*e.g.*, to *“sweat out the poison”*) at the same period (7), it is clear that this first player-driven idea shifted to a more likely coach-driven intervention. Supporting this shift, morning skate became prevalent in the 1970s when it was employed by the Soviet Red Army team during the 1972 Summit Series and subsequently copied with success by the Philadelphia Flyers who won the Stanley Cup in 1974 and 1975 (8). This pioneering practice was eventually implemented league-wide in the NHL, which concurred also with the introduction of new skates that required more maintenance and fine-tuning by the players.

Since then, the morning skate became a pre-game routine/ritual/tradition for most ice hockey players, with some (veteran) players defending its usefulness mainly based on superstitious behavior and/or pre-performance psychological routine (5, 9). More contemporary reasons favoring the morning skate is the lack of time for training and/or tactical work given the high competition density of an ice hockey season with games every 2–3 days (9, 10), with some coaches aiming to take advantage of this extra time to make their teams ready to play (11). The disadvantage of such approach is that it is energy-consuming (*e.g.*, players need to commute, warm up off-ice before going on the ice) and it impairs recovery, thereby being counterproductive in a competitive context that requires to perform with a high frequency (several games per week) throughout the season while being affected by travel-induced jet lag disorder (12, 13). With highly demanding regular seasons and playoffs, more and more ice hockey teams are inclined to cut out game-day morning skate sessions. The 2017 in-season sixteen-game winning streak by the Columbus Blue Jackets, who publicly announced their decision not to use morning skate, may be seen as an incentive example for other squads (14).

Overall, by looking into the supposedly predictable benefits (*e.g.*, extra tactical practice or physical training, familiarization to the visiting ice rink) and drawbacks (extra workload or fatigue) of the morning skate, sports journalists [*e.g.,* (14, 15),] anecdotally reports that teams are currently de-emphasizing or even eliminating the mandatory game-day morning skate, with optional session being the growing trend (*i.e.*, more than a third of NHL teams make some or all morning skates optional).

## Related scientific evidence?

### Superstitious/psychological behavior

Numerous world-class athletes (*e.g.*, Michael Jordan in basketball, Patrick Roy in NHL) have demonstrated superstitious beliefs that wearing particular clothing or carrying out certain pre-shot acts may be followed by a successful outcome, which then reinforces the superstitious behavior (5). When maintained as a “habit” (16), superstition-linked expectancy is accompanied by mental conditioning. These become therefore pre-performance psychological routines defined as “a sequence of task-relevant thoughts and actions which an athlete engages in systematically prior to his or her performance of a specific sports skill” (17, 18). However, their timing immediately prior to execution (*e.g.*, Rafael Nadal before serving in tennis or Jonny Wilkinson before kicking a penalty in rugby union) and their underlying psychophysiological processes (*e.g.*, cardiac deceleration, neural/cognitive function) are not compatible with the time frame between a morning skate and an evening game.

### Training opportunity or delayed potentiation effect?

As previously mentioned, the contemporary use of morning skate by practitioners serves as a pre-game “activation” (or “muscular wake-up”) or an additional training opportunity. Given the timing, the latter remains unlikely, except perhaps for players with lower playing time (*e.g.*, the fourth liners) or for tactical work (*e.g.*, special teams such as power play and penalty killing) that may be performed at lower intensity than classical training sessions. Alternatively, microdosing (*i.e.*, smaller daily training doses but at a higher weekly frequency) (19, 20) or shock microcycle (*i.e.*, higher number of high-intensity sessions within a shorter period lasting 7–14 days) (21, 22) have been proposed to counteract the generally reported in-season physiological detraining effect (23, 24), but these interventions must be carefully considered according to each individual player's needs and team's periodization.

Using the morning skate to promote warm-up-induced neural (*i.e.*, post-activation potentiation) mechanisms (25) is also unlikely due to the possible induction of undesired fatigue (*e.g.*, morning skate sessions induce a ∼34% increase in training load that corresponds to 12 extra matches per season in the American Hockey League, considered as the NHL antechamber) (10) and the short transition phase of post-activation potentiation (*i.e.*, not longer than 18.5 min) (26). While it remains a psychological opportunity to mentally prepare for the upcoming evening game, whether a possible “delayed potentiation” effect exists after morning skate is still unknown. Low-volume and moderate-to-high-intensity resistance or (resisted) sprinting exercise stimulus has shown promising benefits to induce neuromuscular priming for upper- and lower-body performance measured between 1 and 48 h afterwards (4, 27). Considering that priming strategies may have an enhanced effect with sport-specific movements, without excluding possible muscle damage and residual fatigue (high individual variation) in a condensed schedule, the current recommendations suggest implementing a priming session in the morning of a game day—notably for its diurnal effect (*i.e.*, change in testosterone and cortisol concentrations) on player readiness (28)—or the day before, 24–33 h prior to the game (4, 29). Several successful priming interventions have been reported in team sports [*e.g.*, in rugby union (28, 30, 31), rugby sevens (32), soccer (33) and volleyball (34)] but with different response kinetics due to the multiple exercise modes and priming protocols employed. Regarding ice hockey, apart a 4-min post-activation potentiation effect following on-ice heavy resisted skating sprint (35) and a 6-h delayed potentiation resulting from an off-ice contrast training (36), specific on-ice and/or off-ice priming interventions have not yet been scientifically explored.

### Chronobiology, sleep and recovery

Given its relationship to the circadian rhythm and its biological and hormonal responses (37), the timing of a priming session is crucial for an evening game performance. In addition, consideration is required for exogenous factors such as jet lag and consecutive fatigue and sleep disturbance resulting from the extensive travel schedule of NHL players, for instance, which often involves multiple cross-continental flights and back-to-back games in different time zones that can desynchronize the circadian rhythm and consequently the endogenous body-clock component (2). Because such circadian misalignment, regardless of travel direction, affects NHL players' performance (13), considering players' chronotype (*i.e.*, “larks” or morning types have preference for morning activities, while “owls” or evening types prefer afternoon activities) (38, 39) and individual magnitude in diurnal variation (40) would be recommended, in addition to fatigue monitoring, before suggesting additional morning skate or recovery processes and their optimal timing. Interestingly, by eliminating “morning shoot around” in favor of extra sleep or rest (41), an improvement in players' in-game performance has been reported in team sports such as basketball (42). Finally, in addition to increasing sleep opportunity, letting players free on some game-day mornings allows them to refresh mentally or take personal free time (*e.g.*, with family). Increasing such mental wellbeing is also a key component to optimize elite sport performance (43).

## Practical tips

Beside scientific evidence, the decision for having a mandatory or an optional morning skate is dependent on several factors including the teams' schedule (*e.g.*, full practices the day before and then optional morning skate on game day or inversely) and periodization. Then, generational (*i.e.*, veteran players have been formatted to morning skate and need them to feel ready likely based on superstitious beliefs) and personal preferences (*e.g.*, duration, content) also play a role. While the influence of experienced players may provide a positive role model on the motivation and attitude of younger players, it may also have counterintuitive side effects, such as promoting wrong beliefs and behaviors that are not supported by current evidence, thereby catalyzing for example ongoing participation in morning skate.

Players' individual playing time in competition is an important point of consideration as in-season training content has been shown inefficient to maintain fitness levels over a season (23, 24). As such, players with lower playing time (*e.g.*, fourth-liners and healthy scratches) need more training time while “big-minute” players (*i.e.*, with higher playing time) are requested to stay away from the rink as much as possible. Finally, given the substantial inter- and intra-individual variability in response to a training stimuli (1), allowing or advising players to choose between several available alternative options (*e.g.*, skate-, resistance- or sprint-based priming sessions, tactical video analysis, recovery and medical care) can give them a degree of autonomy, boosting motivation and self-confidence.

In all cases, the implementation of morning skate is team-, context- and player-dependent. Therefore, game-day preparation strategies may consider the following factors:

- Competition calendar and density of schedule,
- Previous days (training or not, travel or not) and game time,
- Players' chronotype, habits and preferences,
- Individual on-ice playing time/workload (*e.g.*, low *vs*. high playing time players) and status (fatigued, injured),
- Technical staff needs (*e.g.*, opponent-specific tactical preparation),
- Off-ice alternatives [*e.g.*, meetings, video sessions, resistance or (resisted) sprinting exercise].

**Figure 1:**
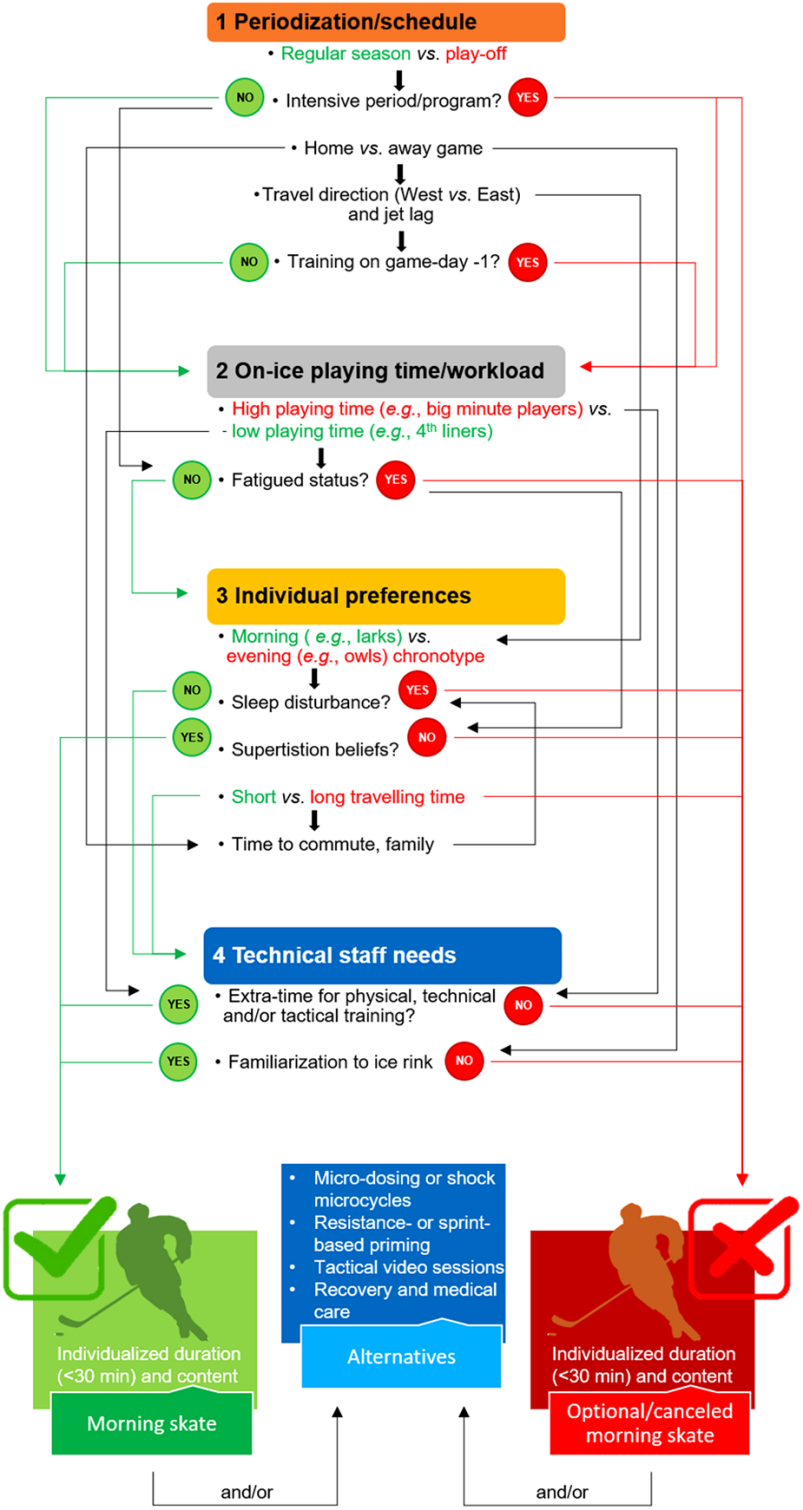
Decision tree for the implementation of morning skate. Green text and arrows refer to a positive weight (or effect); red text and arrows refer to a negative weight (or effect); black arrows branch off into other possible factors.

A decision tree has been proposed to improve the informed decision process for programming a morning skate (Figure 1), even though there is no “one-size-fits-all” solution. It allows practitioners to weigh possible team-, context- and player-dependent factors against one another to drive informal discussion among the technical staff or to map out the best choice to implement or cancel a compulsory or optional morning skate and/or possible alternatives.

## Conclusion

To skate or not on game day has been a question for several decades now. However, optional morning skate is becoming the new norm in major ice hockey leagues as well as “morning shoot around” or other “activation” sessions in other team sports. The reasons for this change are diverse and team-, context- and player-dependent. Thus, in the absence of clear scientific evidence and pending confirmation regarding a possible on-ice priming effect, no general recommendations can be made regarding whether or not to implement morning skate on game day. In the meantime, practitioners may use the points considered in this opinion to take context-specific and informed decisions on the implementation of their game-day preparation strategies.
